# HU308, A Selective Cannabinoid Type-2 Receptor Agonist, Mitigates SARS-CoV-2 Spike Protein–Induced Acute Lung Injury in Mice

**DOI:** 10.1007/s00408-026-00870-6

**Published:** 2026-02-09

**Authors:** Janette Lockett, Gregory Nicholson, Nicholas Richards, Ryan Washington, Nagaraja Nagre

**Affiliations:** Department of Biomedical and Translational Sciences, Macon & Joan Brock Virginia Health Sciences, Eastern Virginia Medical School at Old Dominion University, Norfolk, VA 23507 USA

**Keywords:** Acute lung injury, SARS-CoV-2 spike protein subunit 1, Cannabinoids, Cannabinoid-2 receptor

## Abstract

**Introduction:**

The coronavirus disease 2019 (COVID-19) pandemic, caused by severe acute respiratory syndrome coronavirus 2 (SARS-CoV-2), continues to pose major health challenges despite effective vaccination efforts. The sustained occurrence of breakthrough infections and emerging variants of the virus highlights the need for additional therapeutic strategies. Given the anti-inflammatory role of the cannabinoid type 2 receptor (CB2R), we examined the effect of CB2R activation in SARS-CoV-2 spike protein subunit 1 (S1SP)-induced acute lung injury (ALI).

**Methods:**

ALI was induced in mice by intratracheal (i.t.) administration of S1SP, followed by treatment with the CB2R agonist HU308 (5 mg/kg, intraperitoneal: i.p.) 1 h post-S1SP and every 24 h thereafter. Lung function, bronchoalveolar lavage fluid (BALF) parameters, cytokine levels, and inflammatory signaling were assessed at 48 h following S1SP exposure.

**Results:**

HU308 treatment significantly reduced S1SP-induced pulmonary dysfunction, immune cell infiltration, neutrophil activation, and proinflammatory cytokine production, while suppressing NF-κB and STAT3 activation. HU308 treatment restored the Nrf2 expression in the lung.

**Conclusion:**

CB2R activation ameliorates S1SP-induced lung inflammation and injury, suggesting its therapeutic potential against COVID-19-related ALI.

**Supplementary Information:**

The online version contains supplementary material available at 10.1007/s00408-026-00870-6.

## Introduction

The coronavirus disease 2019 (COVID-19) pandemic was caused by the severe acute respiratory syndrome coronavirus 2 (SARS-CoV-2) that emerged during the winter of 2019. Since then, it has infected over 700 million people worldwide and resulted in more than 7 million deaths. The pandemic has severely impacted global healthcare systems and economies [1, 2].

COVID-19 predominantly affects the respiratory system, with pneumonia being the most prevalent consequence, followed by acute respiratory distress syndrome (ARDS) [2, 3]. The virus induces a hyperinflammatory response in the pulmonary tissues, marked by the overactivation of immune cells. This uncontrolled immune response results in the overproduction of inflammatory cytokines, often referred to as a “cytokine storm”, which can lead to multi-organ failure and death. The severity of COVID-19 is closely associated with the acute overproduction and uncontrolled release of pro-inflammatory cytokines such as interleukin-6 (IL-6), interleukin-1 (IL-1), interleukin-17 (IL-17), and tumor necrosis factor-alpha (TNF-α), both locally and systemically [4, 5]. Although prophylactic vaccines for COVID-19 have demonstrated safety and efficacy in reducing the risk of severe disease, hospitalization, and death, challenges such as vaccine hesitancy, variability in vaccine efficacy, breakthrough infections, and the emergence of new variants highlight the ongoing need for therapeutic interventions that can provide robust protection against severe COVID-19.

The mammalian endocannabinoid system (ECS) is composed of cannabinoid receptors, endocannabinoids such as anandamide (AEA) and 2-arachidonoylglycerol (2-AG), as well as the enzymes responsible for their synthesis and breakdown. AEA and 2-AG exert their biological effects primarily by binding to two types of G-protein-coupled receptors: CB1 receptors (CB1R) and CB2 receptors (CB2R). CB2R is mainly found in the peripheral immune system, and the activation of CB2R exhibits anti-inflammatory and anti-fibrogenic effects without eliciting any adverse psychotic effects [6, 7]. Consequently, comprehending the function of CB2R in immune regulation has been garnering therapeutic attention. Pharmacological activation of CB2R in the lungs has been shown to exhibit a protective effect on murine models of acute and chronic lung injury [8, 9]. Consistent with these findings, our laboratory has demonstrated that activation of CB2R attenuates ALI induced by bacterial pneumonia as well as vesicant exposure in murine models [10, 11]. These results support the potential of CB2R as a therapeutic target for mitigating immune-mediated pathological conditions, including those associated with viral infections.

In this study, we employed a mouse model of ALI induced by the SARS-CoV-2 spike protein subunit 1 (S1SP), a well-established approach that mimics key pathological features of COVID-19, including the “cytokine storm” [12–14]. Considering the anti-inflammatory role of CB2R, we aimed to investigate its potential protective effects in mitigating S1SP-induced ALI. Our findings demonstrate that activation of CB2R using the selective synthetic agonist HU308 effectively attenuates S1SP-induced lung injury and inflammation, reduces neutrophil infiltration, and mitigates excessive neutrophil activation. Furthermore, CB2R activation inhibited the activation of NF-κB (nuclear factor kappa B), and STAT3 (signal transducer and activator of transcription 3) pathways implicated in inflammatory responses. Notably, CB2R activation concomitantly upregulated Nrf2 (nuclear factor erythroid 2-related factor 2) expression.

## Materials and Methods

### Animals and Animal Procedures

Transgenic mice expressing the human (h) ACE2 controlled by the human keratin 18 (K18) promoter (K18-hACE2) were purchased from the Jackson Laboratory and housed in a sterile, ventilated facility at Old Dominion University (ODU) under standard husbandry. Male mice, 8–10 weeks of age, were used in this study. The Institutional Animal Care and Use Committee of ODU approved all the procedures.

S1SP-induced acute lung injury was modeled by intratracheal (i.t.) injection of S1SP (Ray Biotech, 230–01101) into mice at a dose of 0.5 mg/kg (50 µl) using a micro sprayer (Penn Century Inc.) under anesthesia with ketamine and xylazine. The control mice received 50 µl PBS via i.t. injections. CB2R agonist HU308(3088, Tocris Biosciences) was dissolved in a vehicle (1% DMSO + 1% Tween-80 in PBS). To activate CB2R, HU308 (5 mg/kg) was administered to mice via the intraperitoneal (i.p.) route, 1 h after S1SP administration, and every 24 h after that. Mice were distributed into the following groups: (1) vehicle + PBS, (2) HU308 + PBS, (3) vehicle + S1SP, (4) HU308 + S1SP. ALI was assessed at 48 h following S1SP exposure.

### Measurement of Lung Mechanics

Lung mechanics were measured using the FlexiVent system and FlexiWare software (SCIREQ, Montreal, Canada). After 48 h following S1SP exposure, anesthetized mice were intubated intratracheally with an 18 1/2 G catheter and ventilated at a lower tidal volume (10 ml/kg) and 150 breaths/minute for 10 min. Tissue dampening (G) and static lung compliance (Cst) were assessed using FlexiWare software, according to manufacturer recommendations.

### Analysis of Bronchoalveolar Lavage Fluid (BALF)

After measuring lung mechanics, the lungs of mice were lavaged with 3 ml of PBS (3 × 1 ml). The total cell number in the BALF was determined using an automated cell counter (Countess II FL, ThermoFisher Scientific). For differential cell count analysis, BALF cells were cytocentrifuged onto glass slides and subsequently subjected to differential staining using the Kwik–Diff™ kit (Thermo Fisher Scientific) following the manufacturer’s protocol. The total protein content in the BALF was measured using the BCA Protein Assay Kit (Bio-Rad Laboratories). Cytokine levels, specifically IL-6, TNF-α, and IL-10 in the BALF were quantified by ELISA (R&D Systems).

#### Lung Tissue Processing

After collecting the BALF, the right mainstem bronchus was tied off with a 4–0 silk suture, and the right lung was cut and snap-frozen in liquid nitrogen and stored at − 80 °C until further use. The left lung was inflated with 4% paraformaldehyde at 20 cm H_2_O and fixed overnight at 4 °C. The left lobe was processed for paraffin embedding and tissue sectioning.

#### Isolation of Mouse Primary Alveolar Macrophages (AMs)

Mouse AMs were isolated as previously described [11, 15]. Following S1SP exposure, BALF was collected from the mice by lavaging the lungs with 3 ml of PBS, 1 ml at a time. The collected cell suspension was centrifuged at 1000 rpm for 10 min, at 4 °C, and the resulting pellet was resuspended in Dulbecco’s Modified Eagle’s Medium (DMEM) supplemented with 10% non-heat inactivated fetal bovine serum (FBS) and 1% penicillin-streptomycin (P/S). BALF cells were plated in a complete DMEM medium for 3 h, followed by extensive washing with PBS to remove unattached cells. The attached cells were identified as AMs. These cells were harvested by gentle scraping and subsequently used for immunoblot analysis.

#### Isolation of Mouse Primary Alveolar Epithelial Cells

Primary alveolar epithelial cells were isolated from mouse lungs using a previously described protocol [16]. Briefly, following BALF collection, lung tissue was digested with dispase (1.8 U/mL) and passed through a 100-µm mesh filter. Leukocytes were depleted by panning on IgG-coated dishes. The resulting nonadherent cells were sequentially incubated with anti-mouse CD90 antibody to remove fibroblasts, followed by anti-mouse CD31 antibody conjugated to Dynabeads to isolate endothelial cells. The CD31-negative fraction, enriched for a mixture of type I and type II alveolar epithelial cells, was collected and processed for immunoblot analysis.

#### Immunostaining

Paraffin-embedded lung Section (5 μm) were deparaffinized, hydrated, and subjected to antigen retrieval. The sections were stained for neutrophil elastase (NE) using an anti-NE antibody (1:200, ab68672, Abcam) and for H2B using anti-H2B (1:100, ab52484, Abcam). Sections were then incubated with fluorochrome-labeled species-specific secondary antibodies, and the resultant stained sections were imaged using an Olympus IX73 fluorescent microscope. Three to six mice from each group were used in this experiment.

#### Immunoblot Analysis

Lung tissues or cells were lysed in Pierce™ IP Lysis Buffer (ThermoFisher Scientific). The lysates were centrifuged at 12,000 rpm for 15 min at 4 °C. The protein concentration in the supernatant was determined by the BCA Protein Assay (Bio-Rad Laboratories). For the immunoblot, 40 µg of the total protein was resolved in SDS-PAGE and electroblotted onto a polyvinylidene fluoride membrane. The membrane was blocked with 5% non-fat milk in PBS containing 0.1% Tween-20 for 30 min at room temperature. The membrane was incubated with primary antibodies overnight at 4 °C, followed by incubation with secondary antibodies. The membrane was then developed using ECL Western Blot Substrate (ThermoFisher Scientific). The following primary antibodies were used: anti-IκB (4812, 1:1000), anti-P-IκB (9246, 1:500), anti-Stat3 (9139, 1:500), anti-P-Stat3 (9145, 1:500) from Cell Signaling Technology Inc., anti-peptidylarginine deiminase 4 (PAD4) (1:500), anti-CB2R (1:500) from Abcam, and anti-β-actin (1:5000, A5441, Sigma-Aldrich). For analysis of Nrf2 expression, nuclear extracts were prepared from lung tissue using the NE-PER™ Nuclear and Cytoplasmic Extraction Reagents (ThermoFisher Scientific) according to the manufacturer’s instructions. The nuclear fractions were subsequently processed for immunoblotting using anti-Nrf2 (1:1000, NBP1-32822, Novus Biologicals). For immunoblot analysis of BALF samples, equal volumes of BALF from each experimental group were subjected to SDS-PAGE and transferred onto PVDF membranes. The membranes were probed with the following primary antibodies: anti-Histone H2B (1:500; ab52484, Abcam) and anti-citrullinated histone H3 (CitH3) (1:500; ab5103, Abcam).

##### Statistical Analysis

All results are presented as the mean ± standard error of the mean (SEM). Statistical comparisons were made using Prism 10.4.2 (GraphPad Inc). The group differences were analyzed using Student’s t-test or one-way ANOVA, followed by a Tukey’s post-hoc multiple comparison tests when appropriate. Differences were considered statistically significant at *p* < 0.05.

## Results

### CB2R Activation Lowers S1SP‑induced Lung Injury and Inflammation

**Fig. 1:**
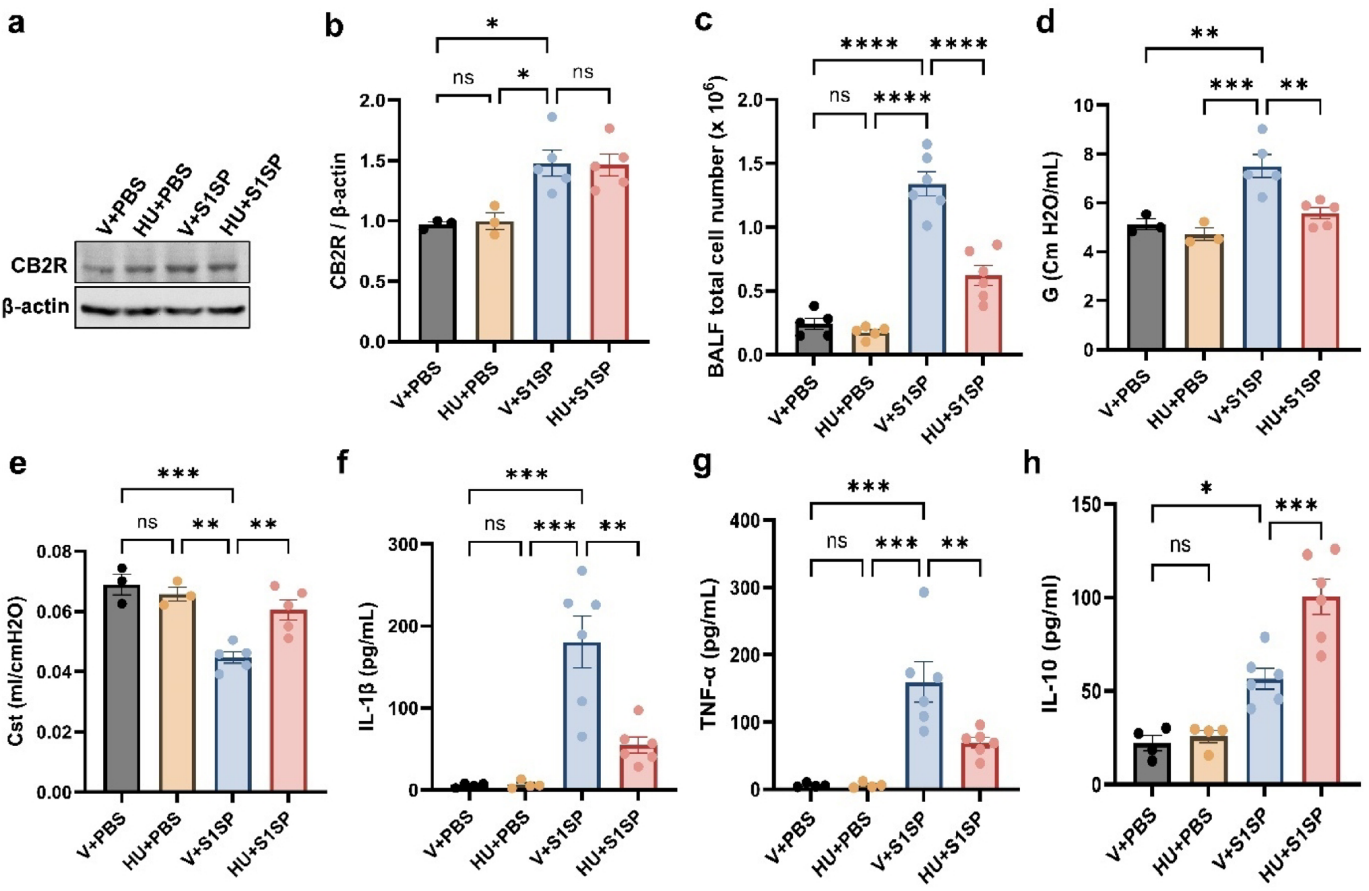
HU308 treatment alleviates S1SP-induced lung injury and inflammation. K18-hACE2 mice were exposed to PBS (control) or S1SP (0.5 mg/kg) and treated with vehicle or HU308. CB2R expression in AMs was assessed at 48 h following S1SP exposure. The representative immunoblots images are shown in panel (**a**), and densitometry data are presented in panel (**b**). The total cell number (**c**) in BALF was determined at 48 h post-S1SP exposure. (**d**) Tissue dampening (G) and (**e**) static lung compliance (Cst) were examined using the FlexiVent system. The levels of inflammatory cytokines IL-1β (**f**) and TNF-α (**g**), and anti-inflammatory cytokine IL-10 (**h**) were measured. *n* = 3–6, * *p* < 0.05, **p < 0.01, ***p < 0.001, ****p < 0.0001. Data are presented as mean ± SEM. V: vehicle, HU: HU308

To define the cellular targets of CB2R signaling in the lung following S1SP exposure, we assessed CB2R expression in AMs and alveolar epithelial cells. CB2R expression was markedly upregulated in AMs after S1SP exposure (Fig. 1a, b). In contrast, CB2R levels in alveolar epithelial cells were low at baseline and did not change following S1SP (Supplementary Fig. 1a, b). The infiltration of immune cells into the lung was assessed by measuring the total cell number in the BALF. S1SP exposure significantly increased BALF total cell numbers, while activation of CB2R with HU308 markedly attenuated this increase (Fig. 1c). HU308-treated mice also exhibited reduced BALF protein contents, a marker for lung microvascular permeability (Supplementary Fig. 2). Assessment of lung mechanics using the FlexiVent ventilator system demonstrated that S1SP exposure resulted in increased parenchymal resistance (G) and decreased static lung compliance (Cst), indicative of impaired lung function. HU308 treatment significantly alleviated these alterations in lung mechanics (Fig. 1d, e). Proinflammatory cytokines, such as IL-1β and TNF-α, are potent mediators of inflammation and are associated with the severity of COVID-19 [17]. Here, we observed that S1SP exposure led to a significant increase in the levels of IL-1β and TNF-α (Fig. 1f, g) in the BALF. Interestingly, HU308-treated mice had significantly lower levels of these cytokines. In contrast, CB2R activation by HU308 markedly increased the levels of the anti-inflammatory cytokine IL-10 in the BALF (Fig. 1h).

**Fig. 2:**
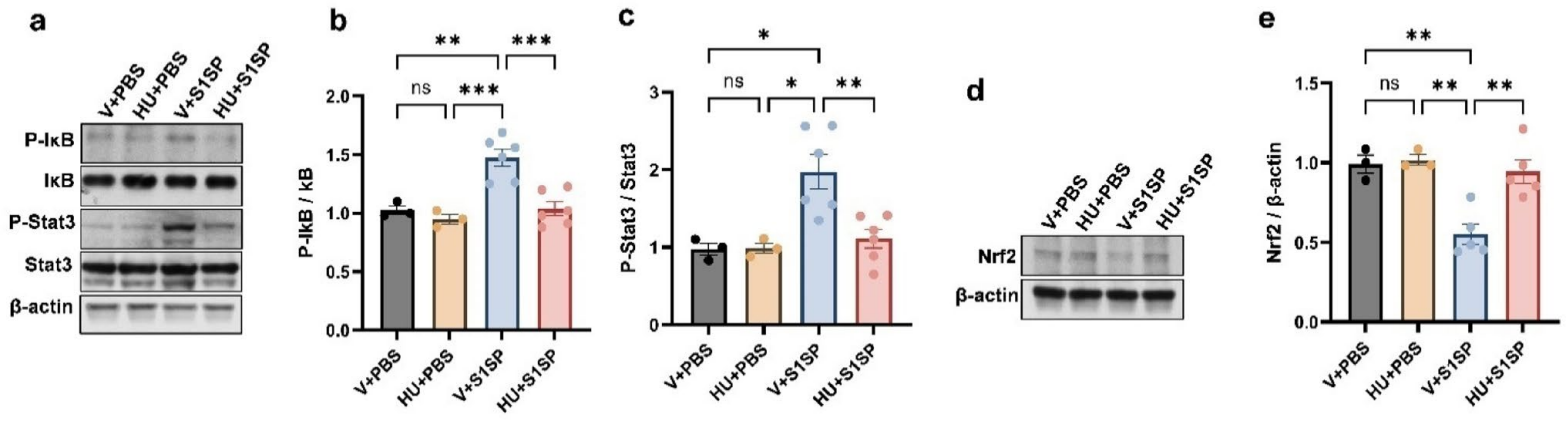
CB2R activation attenuates S1SP-induced inflammatory signaling in the lung. K18 hACE2 mice were exposed to PBS or S1SP (0.5 mg/kg) and treated with vehicle or HU308. Lung tissue was collected after 48 h. NF-κB activation was assessed by measuring the protein levels of P-IκB and IκB in the lung. STAT3 activation was examined by measuring the protein levels of P-Stat3 and Stat3 in the lung. Representative immunoblot images are shown in panel (**a**), and densitometry data are presented in panels (**b**) and (**c**). Representative immunoblot (**d**) and densitometric analysis (**e**) of Nrf2 protein levels in nuclear extracts isolated from lung tissue. β-actin was used as a loading control throughout. *n* = 3–6, *p < 0.05, **p < 0.01. Data are presented as mean ± SEM. V: vehicle, HU: HU308

To investigate the role of CB2R activation on NF-κB signaling in S1SP-induced inflammation, we examined the phosphorylation status of IκB (P-IκB), a marker of NF-κB activation, in lung tissues. Immunoblot analysis revealed elevated P-IκB levels following S1SP exposure, indicating enhanced NF-κB activity. HU308 treatment significantly reduced P-IκB levels (Fig. 2a, b). Similarly, levels of phosphorylated Stat3 (P-stat3) were significantly lower in HU308-treated mice compared to vehicle-treated controls post-S1SP exposure (Fig. 2a, c). These findings suggest that CB2R activation attenuates S1SP-induced lung inflammation by downregulating NF-κB and Stat3 signaling pathways. To further delineate the mechanism underlying the anti-inflammatory effects of HU308, we assessed nuclear Nrf2 protein expression in lung tissue in parallel with NF-κB activation. Immunoblot analysis revealed that S1SP exposure markedly suppressed nuclear Nrf2 levels. Notably, treatment with the CB2R agonist HU308 significantly restored nuclear Nrf2 expression toward baseline levels (Fig. 2d, e). Collectively, these findings suggest that CB2R activation counteracts S1SP-mediated inhibition of Nrf2, and that restoration of Nrf2 signaling may contribute to the attenuation of NF-κB-driven inflammatory responses observed following HU308 treatment.

### HU308 Treatment Reduces Neutrophil Infiltration and NET Formation

**Fig. 3:**
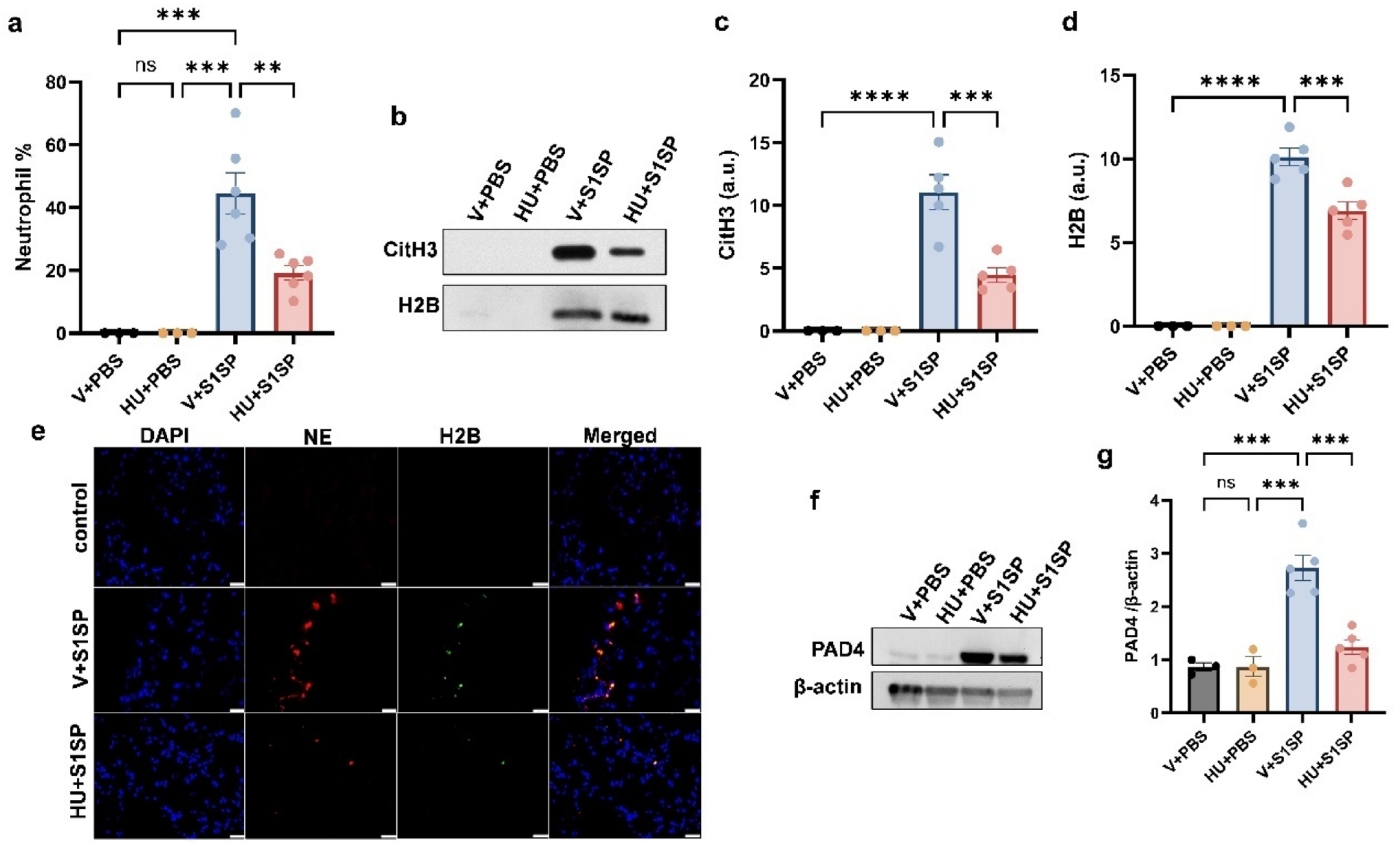
HU308 treatment reduces neutrophil infiltration and activation. K18 hACE2 mice were exposed to PBS or S1SP (0.5 mg/kg) and treated with vehicle or HU308. BALF was collected after 48 h, and the neutrophil population (**a**) was assessed by differential staining. The release of CitH3 and H2B in the BALF was examined by immunoblot. Representative immunoblot (**b**) and quantification of CitH3 (**c**) and H2B (**d**) in BALF from mice exposed to PBS or S1SP and treated with vehicle or HU308. (**e**) Representative Immunostaining image of NE (red) and H2B (green) in mouse lungs exposed to PBS or S1SP and treated with vehicle or HU308. The level of PAD4 in the lung was examined by immunoblot. Representative immunoblot images are shown in panel (**f**), and densitometry data are presented in panel (**g**). *n* = 3–6, *p < 0.05, **p < 0.01, ***p < 0.001, ****p < 0.0001. Data are presented as mean ± SEM. V: vehicle, HU: HU308, a.u: arbitrary units

Neutrophil activation, including the release of neutrophil extracellular traps (NETs), is a critical pathological mechanism underlying ARDS, particularly in severe COVID-19 cases. In this study, analysis of BALF cells using differential staining revealed a significant reduction in neutrophil populations in HU308-treated mice compared to vehicle-treated controls following S1SP exposure (Fig. 3a). To further examine NET formation, we assessed extracellular histones, including Citrulline-H3 and Histone-H2B, using immunoblot analysis. S1SP exposure markedly elevated Citrulline-H3 and Histone-H2B levels in BALF, whereas HU308 treatment significantly attenuated these increases (Fig. 3b–d). We evaluated the expression of NE and H2B in lung tissues by immunofluorescence analysis. CB2R activation attenuated the expression of both NE and H2B (Fig. 3e). In addition, we assessed the expression of PAD4, a key enzyme mediating NET formation. Notably, CB2R activation significantly suppressed the S1SP-induced upregulation of PAD4 (Fig. 3f–g). These results collectively demonstrate that CB2R activation effectively suppresses S1SP-induced neutrophil infiltration and excessive neutrophil activation. Importantly, the reduction in NET-associated markers closely paralleled the decrease in lung neutrophil numbers, suggesting that the diminished NET signal may be due to reduced neutrophil accumulation rather than a direct neutrophil-intrinsic inhibition of NET formation.

## Discussion

In this study, we showed that CB2R activation with the potent agonist HU308 significantly alleviated S1SP-induced acute lung injury and inflammation by suppressing NF-κB, STAT3, and neutrophil activation. Importantly, HU308 treatment also restored Nrf2 signaling in lung tissue, which was markedly suppressed following S1SP exposure. Using an S1SP mouse model designed to explore COVID-19 therapies, we demonstrated that intratracheal administration of S1SP (0.5 mg/kg) induced reproducible lung injury.

The endocannabinoid system comprises endocannabinoids, cannabinoid receptors (CB1R and CB2R), and enzymes regulating their synthesis and degradation [6]. CB2R, primarily expressed in immune cells, plays a crucial role in immunomodulation, and selective CB2R agonists are of considerable therapeutic interest [9–11]. The lung injury observed at 48 h after S1SP exposure was reduced by CB2R activation. The i.t. administration of S1SP increased the BALF total cell counts; however, HU308 treatment significantly reduced them, indicating a diminished influx of immune cells into the lung. CB2R activation by HU308 significantly reduced pro-inflammatory cytokines IL-6 and TNF-α in BALF, key drivers of cytokine storms in severe COVID-19. Importantly, HU308 treatment also significantly increased BALF levels of the anti-inflammatory cytokine IL-10, suggesting that CB2R activation not only dampens pro-inflammatory signaling but also promotes resolution of inflammation.

Consistent with previous mechanistic studies [14], S1SP exposure induced activation of NF-κB and STAT3, reflecting their established roles in mediating inflammatory responses in the lung. Notably, HU308 treatment significantly attenuated the activation of both these inflammatory pathways. In parallel, we observed that S1SP exposure suppressed the Nrf2 signaling in the lung tissue, and importantly, HU308 treatment restored Nrf2 levels. Nrf2 is known to negatively regulate NF-κB signaling through multiple mechanisms, including enhancing antioxidant defenses and stabilizing IκBα [18, 19]. Thus, the restoration of Nrf2 by HU308 provides a plausible upstream mechanism for the reduced phosphorylation of IκB and diminished NF-κB activation observed in our model. This aligns with earlier studies that CB2R signaling intersects with redox-regulatory pathways and may promote anti-inflammatory responses through an Nrf2-dependent mechanism [20, 21].

Among the key drivers of immune dysregulation in COVID-19, neutrophil activation and the release of NETs have garnered significant attention. Elevated NETs have been identified in COVID-19 patients, with SARS-CoV-2 nucleocapsid and spike proteins inducing NET formation [22, 23]. Histones released during NETosis enhance SARS-CoV-2 infectivity [24], while PAD4, an enzyme critical for chromatin decondensation, plays a central role in NET formation [25]. CB2R has been implicated in modulating the function of immune cells in inflammatory diseases. Studies show that CB2R activation suppresses neutrophil recruitment, whereas its absence exacerbates neutrophil-driven inflammation [26]. Research from our lab demonstrated that activation of CB2R reduced NET formation in an ALI mouse model [10]. In the current study, S1SP exposure increased neutrophil infiltration along with elevated levels of Citrulline-H3 and Histone-H2B, markers of NETs. CB2R activation significantly reduced these changes, and treatment with HU308 significantly reduced lung PAD4 levels, which were elevated in response to S1SP exposure. These findings highlight the regulatory role of CB2R in mitigating S1SP-induced neutrophil activation and NET formation. Notably, the concordant decline in NET markers and lung neutrophil burden suggests that CB2R-mediated effects on NETs may be secondary to diminished neutrophil accumulation, rather than a neutrophil-intrinsic blockade of NET formation. Furthermore, we found that CB2R expression was markedly upregulated in AMs following S1SP exposure, whereas CB2R expression in alveolar epithelial cells was low to negligible and unchanged. Together, these findings suggest that CB2R signaling primarily acts on macrophages to shape the inflammatory milieu and limit neutrophil recruitment, thereby indirectly reducing overall neutrophil activation in the lung.

## Conclusion

In conclusion, our findings demonstrate that selective activation of CB2R by HU308 confers protection against S1SP-induced acute lung injury by dampening macrophage-driven inflammatory signaling, suppressing NF-κB and STAT3 activation, restoring Nrf2 signaling, and limiting neutrophil recruitment and activation. Collectively, these data support CB2R as a potential immunomodulatory therapeutic target for mitigating SARS-CoV-2-associated lung injury and excessive inflammatory responses.

## Supplementary Information

Below is the link to the electronic supplementary material.

Supplementary Material 1

## Data Availability

Data supporting the findings of this study are available from the corresponding author upon request.
