# Supplementary material for: HU308, A Selective Cannabinoid Type-2 Receptor Agonist, Mitigates SARS-CoV-2 Spike Protein–Induced Acute Lung Injury in Mice
Source: Lung. 2026 Feb 9;204(1):7. doi: 10.1007/s00408-026-00870-6 (PMC12883501; doi:10.1007/s00408-026-00870-6)
Supplement: Supplementary file 1 — Supplementary Material 1 [file 408_2026_870_MOESM1_ESM.docx]

**
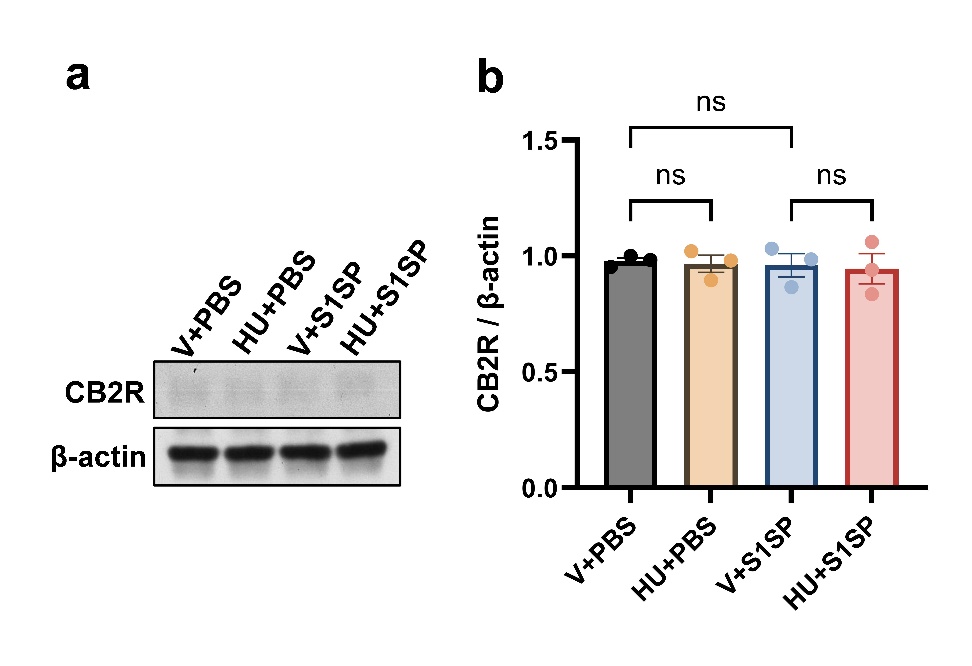
**

**Supplementary Fig. 1.** K18 hACE2 mice were exposed to PBS (control) or S1SP (0.5 mg/kg) and treated with vehicle or HU308. CB2R expression in alveolar epithelial cells was assessed at 48h following S1SP exposure. The representative immunoblots images are shown in panel (a), and densitometry data are presented in panels (b). n=3. Data are presented as mean ±SEM. V: vehicle, HU: HU308. ns: non-significant.


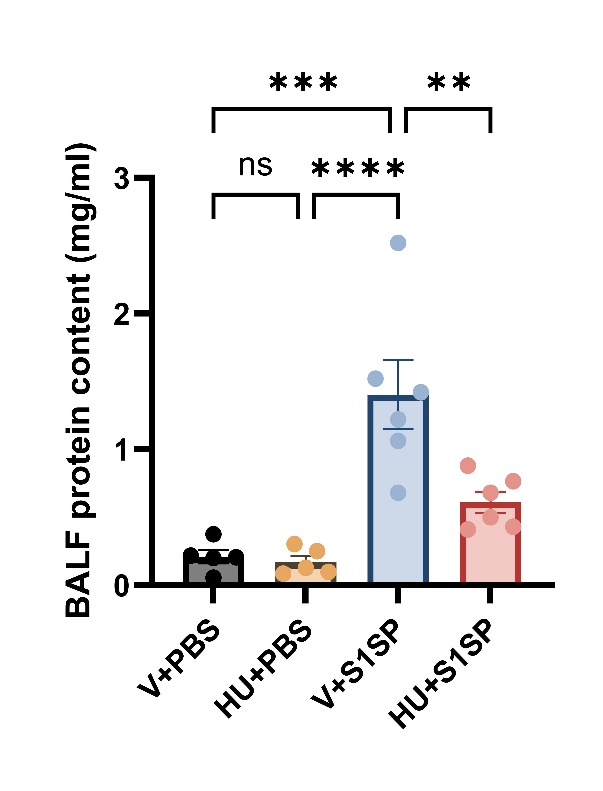


**Supplementary Fig. 2.** HU308 treatment alleviates S1SP-induced lung injury and inflammation. K18 hACE2 mice were exposed to PBS (control) or S1SP (0.5 mg/kg) and treated with vehicle or HU308. The total protein content in BALF was determined at 48h post-S1SP exposure. n=5-6, **p<0.01, ***p<0.001, ****p<0.0001. Data are presented as mean ±SEM. V: vehicle, HU: HU308.
